# Implementation of a pediatric early warning score tool in a pediatric oncology Ward in Palestine

**DOI:** 10.1186/s12913-021-07157-x

**Published:** 2021-10-26

**Authors:** David Mills, Alexis Schmid, Mohammad Najajreh, Ahmad Al Nasser, Yara Awwad, Kholoud Qattush, Michael C. Monuteaux, Joel Hudgins, Zeena Salman, Michelle Niescierenko

**Affiliations:** 1 Boston Children’s Hospital, Boston, USA; 2 Harvard Medical School, Boston, USA; 3 Huda Al Masri Pediatric Oncology Department, Beit Jala, Palestine; 4 Harvard University, Boston, USA; 5 Palestine Children’s Relief Fund, Kent, OH USA

**Keywords:** Quality improvement, Children, Training/education, Workforce and workload, Developing countries

## Abstract

**Background:**

Pediatric Early Warning Scores (PEWS) are nurse-administered clinical assessment tools utilizing vital signs and patient signs and symptoms to screen for patients at risk for clinical deterioration.^1–3^ When utilizing a PEWS system, which consists of an escalation algorithm to alert physicians of high risk patients requiring a bedside evaluation and assessment, studies have demonstrated that PEWS systems can decrease pediatric intensive care (PICU) utilization, in-hospital cardiac arrests, and overall decreased mortality in high income settings. Yet, many hospital based settings in low and lower middle income countries (LMIC) lack systems in place for early identification of patients at risk for clinical deterioration.

**Methods:**

A contextually adapted 16-h pediatric resuscitation program included training of a PEWS tool followed by implementation and integration of a PEWS system in a pediatric hematology/oncology ward in Beit Jala, Palestine. Four PDSA cycles were implemented post-implementation to improve uptake and scoring of PEWS which included PEWS tool integration into an existing electronic medical record (EMR), escalation algorithm and job aid implementation, data audits and ward feedback.

**Results:**

Frequency of complete PEWS vital sign documentation reached a mean of 89.9%. The frequency and accuracy of PEWS scores steadily increased during the post-implementation period, consistently above 89% in both categories starting from data audit four and continuing thereafter. Accuracy of PEWS scoring was unable to be assessed during week 1 and 2 of data audits due to challenges with PEWS integration into the existing EMR (PDSA cycle 1) which were resolved by the 3rd week of data auditing (PDSA cycle 2).

**Conclusions:**

Implementation of a PEWS scoring tool in an LMIC pediatric oncology inpatient unit is feasible and can improve frequency of vital sign collection and generate accurate PEWS scores.

**Contribution to the literature:**

This study demonstrates how to effectively implement a PEWS scoring tool into an LMIC clinical setting.

This study demonstrates how to utilize a robust feedback mechanism to ensure a quality program uptake.

This study demonstrates an effective international partnership model that other institutions may utilize for implementation science.

**Supplementary Information:**

The online version contains supplementary material available at 10.1186/s12913-021-07157-x.

## Background

More than 6 million children around the world die each year, with the majority of these deaths from preventable disease [1]. Almost one-third of deaths under the age of five are due to reversible critical illness such as respiratory failure and sepsis, with children in low- and lower-middle income countries (LMIC) disproportionately affected [2–5]. In hospital-based care, inadequate initial assessment, lack of ongoing monitoring, and inappropriate treatment contribute to poor outcomes, in part due to lack of systems that identify patients at risk for clinical deterioration [6–8].

Pediatric Early Warning Scores (PEWS) are nurse-administered clinical assessment tools utilizing vital signs and patient signs and symptoms to accurately identify patients at risk for clinical deterioration [9–11]. When utilized in a PEWS system which includes an escalation algorithm to alert physicians of high risk patients, studies have demonstrated PEWS systems can decrease PICU utilization, in-hospital cardiac arrests, and overall mortality in high income settings [12, 13]. Yet, many hospital based settings in LMICs lack systems in place for early identification of patients at risk for clinical deterioration.

**Fig. 1:**
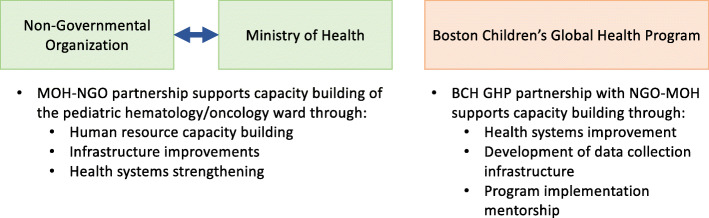
Partnership Framework. Figure demonstrates the partnership framework between the non-governmental organization, the ministry of health, and boston children’s hospital

In 2018, Boston Children’s Global Health Program (BCH GHP) partnered with a Ministry of Health (MOH)-Non-Governmental Organization (NGO) run pediatric hematology/oncology department in the occupied Palestinian territory (oPt)—an LMIC economy [14]—to implement a formal PEWS tool as a component of a pediatric resuscitation training initiative (Fig. 1). There was no previous department-wide training in pediatric resuscitation or indentification of ill appearing patients. We utilized a quality improvement approach to assess the feasibility of implementation of a PEWS tool in a resource limited setting. Our aims were to:

1. Evaluate baseline vital sign documentation with a goal to increase compliance above 80%.
2. Implement a PEWS tool with greater than 80% compliance in frequency and accuracy of PEWS scoring over a three month post-intervention surveillance period.

## Methods

### Setting and context

This study was undertaken in a pediatric hematology and oncology (h/o) ward housed within a general adult and pediatric MOH hospital in Beit Jala, oPt. Patients in the oPt rely on a fragmented health care system defined by severe resource limitations including health care workforce shortages [15]. The ward benefits from an MOH-NGO partnership for technical and operational support. The 14-bed ward admits approximately 100–120 patients per month. There are approximately 75 new cancer diagnoses per year presenting or referred to the department from within the oPt. There is no pediatric intensive care (PICU) on-site, thus any patients requiring critical care, including non-invasive ventilation, mechanical ventilation, or vasoactive therapies require transfer to outside hospitals within the West Bank, including Jerusalem, or hospitals in Israel for further evaluation and management.

The ward team is comprised of an interdisciplinary team that includes one pediatric oncologist, two general practicioners, rotating resident physicians, nurses, one pharmacist, and a ward social worker. Physician coverage includes two physicians during day shifts and coverage by a rotating resident physician during the evening and overnight shift. Rotating resident physicians are also responsible for overnight coverage of the neonatal intensive care unit, general pediatrics ward, and pediatric emergency department. There are 2–3 nurses staffing the ward during the day and one nurse caring for the entire ward during the overnight shift.

### On-site needs assessment

In April 2019, prior to the PEWS intervention, an on-site needs assessment was undertaken by a physician and nurse team from the BCH GHP. The purpose of this assessment was to inform the development of a contextually adapted resuscitation and PEWS program. The needs assessment consisted of nursing, physician, and pharmacy leadership and staff individual interviews and focus groups to identify current ward care practices, resource availability, and expectations. All interviewees were directly involved in clinical care or clinical operations on the ward. The needs assessment discussions were primarily driven by input of participants to identify ward priorities for patient care improvements guided by a list of open-ended questions (Supplemental Document 1).

Needs assessment data were reviewed to identify common themes related to limitations, opportunities, and ward priorities. Common themes during interviews and focus groups (Table 1) included limitations such as high patient volumes, limited resources, staff shortages, and concern for outdated resuscitation protocols. Opportunities for improving care in the ward that were identified included the desire for further training in resuscitation and identification of the ill patient, desire for further basic nursing education including basic vital sign collection, and updating of protocols, policies, code medications, resources, and education. A summary of the thematic content was compiled and presented to the MOH-NGO ward leadership. Feedback from leadership on the identified needs, opportunities, and priorities was incorporated into the development of the PEWS implementation.

**Table 1:** Needs assessment findings

Themes identified during Hematology/Oncology Ward Needs Assessment	
*Limitations*	*Opportunities*
High patient load	Knowledge advancement of identification of the ill child
Resource limitations	Development of resuscitation knowledge/skills
Staff shortages	Knowledge/skills advancement in basic nursing education
Outdated resuscitation protocols	Update of protocols, policies, and code medications resources and education

### Intervention

The intervention consisted of the following components: A pediatric resuscitation course for nurses and phsyicians, PEWS tool didactic and bedside education, integration of the PEWS tool into nursing workflow, and development and integration of an escalation algorithm into the ward workflow.

### Resuscitation training

Ward priorities and requests for resuscitation curriculum content were elucidated prior to and during the needs assessment. Priorities included training for physicians and nurses in contextually adapted pediatric resuscitation and identification of the child at risk for clinical deterioration. In discussion with the nursing leadership, refreshers in basic nursing education, an orientation to the medical code cart, medication mixing, medication administration, and weight based dosing guidelines were all identified or requested. Furthermore, refreshers on pediatric physiology and vital signs collection were core components of training and served as a proxy for ensuring accurate physiologic observations in vital signs collection. The needs assessment and aforementioned priorities informed the development of a contextually adapted 16-h pediatric resuscitation program that included training of PEWS and consisted of a didactic course, hands-on resuscitation skills workshops, and low fidelity simulation for physicians and nurses. Each provider was provided with a resuscitation manual translated to English and Arabic. Instruction was provided by two pediatric emergency medicine physicians and one pediatric emergency nurse with expertise and experience in teaching in pediatric resuscitation. Parallel nursing and physician tracks were developed to tailor content to the respective needs of each discipline (Table 2). Two cohorts of physicians (total *n* = 20), which included physicians from other departments in the hospital, and two cohorts of nurses (total *n* = 12) successfully completed the resuscitation/PEWS course with over 95% attendance from September 1st to 15th 2019.

**Table 2:** Pediatric Resuscitation and PEWS curriculum

Pediatric resuscitation and PEWS-RL curriculum	
*Physician Track*	*Nursing Track*
• Approach to the seriously ill child • Primary and Secondary assessment: evaluation and management • Introduction to PEWS and evidence supporting PEWS effectiveness in clinical practice • Respiratory distress and respiratory failure: recognition and management • Shock: recognition and management • Emergencies in oncology • Basic Life Support (BLS) and Cardiopulmonary Resuscitation (CPR) • Cardiac arrhythmias: recognition and management • Orientation to the code trolley • Practical application of knowledge and skills	• Approach to the seriously ill child: primary and secondary assessment • Obtaining vital signs and vital signs interpretation • Introduction to pediatric early warning scores, evidence supporting PEWS effectiveness in clinical practice, and clinical application • Respiratory distress and respiratory failure: recognition and management • Cardiovascular: anatomy, physiology, assessments; shock recognition and management • Basic Life Support and (BLS) and Cardiopulmonary Resuscitation (CPR) • Emergencies in oncology • Orientation to the code trolley • Practical application of knowledge and skills

### Pediatric early warning score tool and escalation algorithm

A literature review of pediatric early warning scoring tools was undertaken after review of the needs assessment was completed. PEWS-Resource Limited (PEWS-RL), a validated PEWS tool in a resource limited setting [16], was chosen as the PEWS tool to be implemented as the scoring parameters closely match bedside assessment capabilities of the ward compared to other PEWS tools which varied in number and complexity of assessment of scoring components. Six vital signs (heart rate, respiratory rate, respiratory distress, oxygen use, temperature, mental status) of which two are age-adjusted (heart rate and respiratory rate) are required to score PEWS-RL (Supplemental Document 2). An escalation algorithm was developed based on the ward staffing model which outlines the appropriate physician to contact if an elevated PEWS score is obtained.

### Study population, measures and analysis

Inclusion criteria for patients in the study included the following: In the pre-intervention period, all patients admitted to the inpatient ward were included in the study. In the post-intervention period, inclusion criteria for data audits were based on a random interval of data collection every 7–10 days of all patients admitted to the ward on the given day of data collection. Six months of pre-intervention vital signs data (March 1st 2019 to September 1st 2019) were collected to establish the baseline vital signs documentation prior to the intervention. Pre-intervention data included all vital signs (heart rate, respiratory rate, oxygen use, respiratory distress, temperature, mental status) collected during vital sign occurences for all admissions to the ward during the given time interval. A vital sign occurrence is defined as the event where nursing collects vital signs at the bedside, which occurs four times daily. Vital signs are documented in patient charts located in an electronic medical record (EMR). Pre-intervention data was collected using a case report form (CRF) based in KoBoToolbox, an open-source field data collection tool [17]. Three months of post-intevention data collection (September 15th to December 31st 2019) was collected. Random data audits were completed as a feasible real time PEWS feedback mechanism. Data collected included individual vitals signs and ‘complete’ vital sign occurrences. A ‘complete’ vital sign occurrence was defined as all six PEWS-RL vital signs documented during a vital sign collection. Complete vital sign collection should occur three times daily. A fourth vital sign check, which is comprised of a temperature check, is noted as ‘complete’ if the temperature is documented. Outcomes were defined as the change in vital signs frequency between the pre-intervention period and post-intervention data audit period and the PEWS frequency and accuracy in the post-intervention period. PEWS frequency was defined as the percentage of time that PEWS was scored in a given data audit, and accuracy was defined as an accurate PEWS score as verified by a study research assistant calculating PEWS from raw data audit.

### Data compliance and IRB

All data collection was completed on-site in Beit Jala, oPt. Institutional review board approval was obtained through Boston Children’s Hospital and the Palestinian MOH.

### PDSA cycles

#### PDSA #1: PEWS-RL scoring tool EMR integration

The PEWS-RL scoring tool was integrated into the EMR. Initial location for PEWS-RL scoring documentation within the EMR was identified and launched in real time after completion of the resuscitation course. The initial EMR documentation section (within the vital signs tab) that was identified for nurses to document the PEWS-RL score was experiencing technical issues, thus an alternative site in the vital signs documentation location within the EMR was chosen for scoring documentation.

#### PDSA #2: PEWS-RL and job aid implementation

Real-time, shoulder-to-shoulder nursing implementation support was provided by the BCH GHP team comprised of one pediatric emergency nurse and two pediatric emergency medicine physicains.

Job aids including PEWS tool scoring charts, normal vital signs for age, and a ward escalation algorithm were placed in strategic charting locations throughout the ward to facilitate use and documentation of the score in the identified EMR field and activation of the escalation algorithm when indicated.

#### PDSA #3: Data audits to identify compliance in PEWS-RL scoring frequency and accuracy (Fig. 2)

During the post-implementation period (September 16th to December 31th 2019), random data audits completed every 7–10 days were done to assess for vital sign and PEWS scoring frequency as well as PEWS scoring accuracy. These audits were undertaken by a local, Arabic language fluent research assistant who retrospectively collected vital signs and PEWS data on select admitted patients. All data was collected after discharge on the same day via the KoBoToolbox CRF [17]. Review of nursing PEWS scoring accuracy was completed remotely by the BCH GHP implementation team to identify inaccurate PEWS scoring (Table 3). Vital sign completeness and PEWS accuracy and frequency were reported back to the ward physician and nursing leadership. Specific examples of common errors in scoring were noted for potential areas of score improvement.

**Fig. 2:**
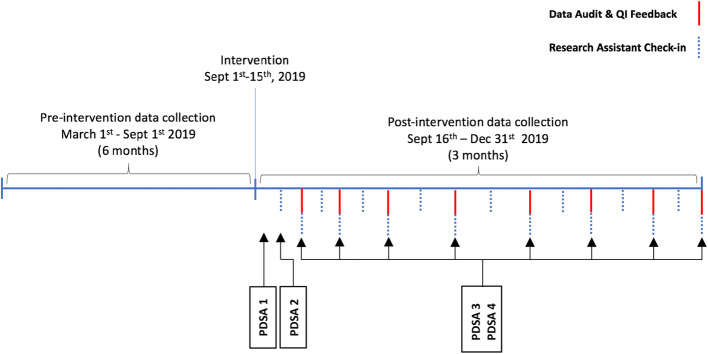
Data collection and PDSA Cycle timelin

**Table 3:** Post-implementation PEWS data audit

Post-Implementation PEWS Data Audit								
Post-implementation week	1	2	3	4	5	6	7	8
Vital sign occurrences audited (n)	44	35	56	43	78	70	65	47
Frequency of PEWS scored (%)	93	94	98	93	96	100	100	97
Accuracy of PEWS score (%)	--*	--*	82	67	89	97	100	89

*Unable to calculate PEWS accuracy

#### PDSA #4: Post-implementation encouragement through data audits (Fig. 2)

Results from data audits were disseminated to ward leadership. Updates were provided to and discussed with nursing and physician staff at morning ward meetings. Ward nursing and physician feedback was solicited, which informed modifications to improve the ease of PEWS scoring, documentation and job aid utilization. Ward QI champion roles were defined and carried out by a physician and nursing leader who demonstrated interest in continuing the QI implementation work.

### Statistical analysis

We used a pre-post cohort design to assess differences in frequency of complete vital signs documentation per vital sign occurrence utilizing statistical process control methodology. For post-intervention data audits, we approximated a 10% data audit to minimize the chance for data sampling bias.

## Results

**Fig. 3:**
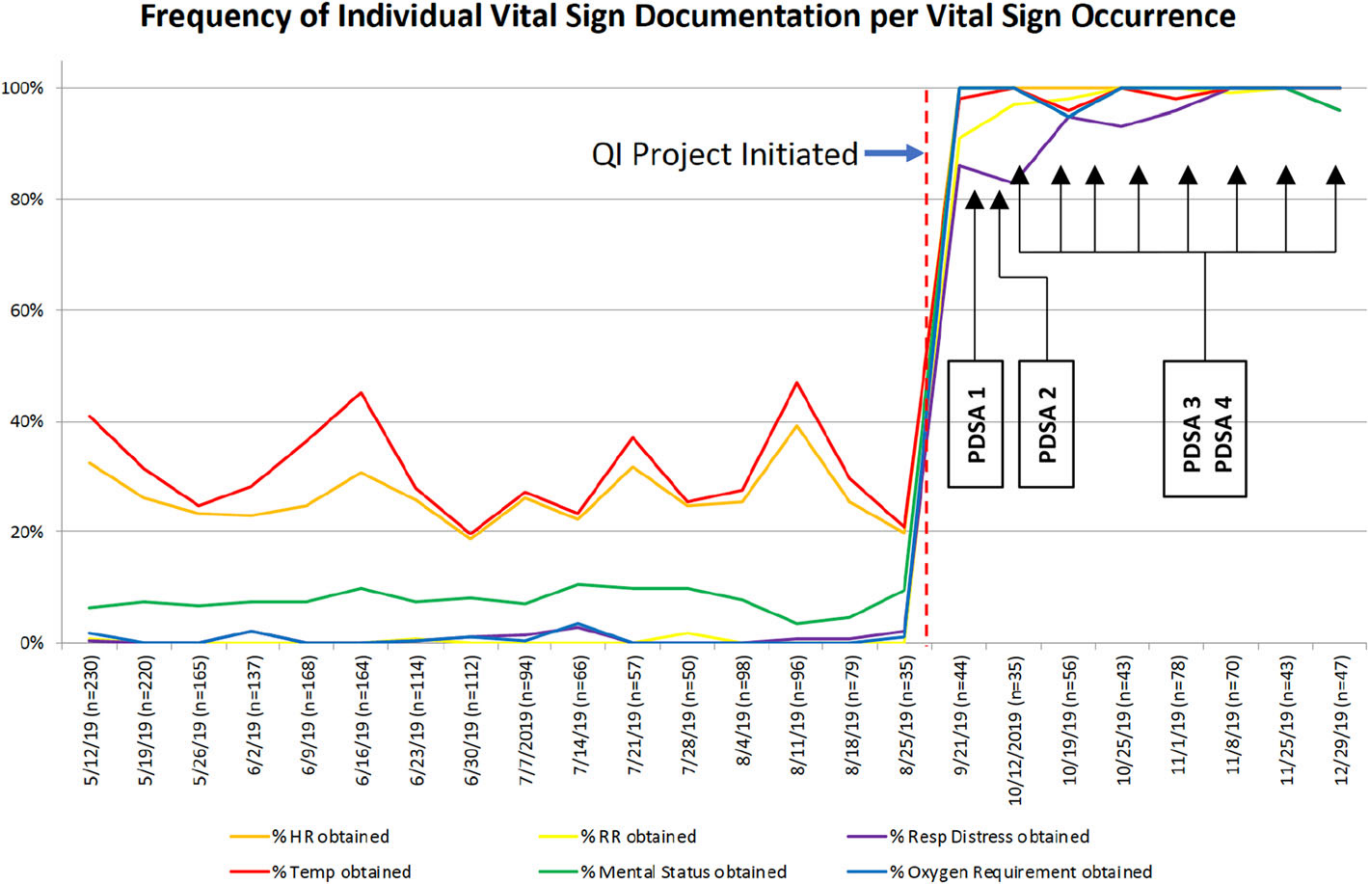
Frequency of Individual Vital Sign Documentation per vital sign occurrence. Figure 3 demonstrates the percentage of individual vital sign documentation by nursing staff per vital sign occurrence. Pre-intervention documentation demonstrates all vital sign documentation below 50% compliance, with four vital signs (respiratory rate, mental status, respiratory distress, oxygen use) consistently below 15%. Post-intervention documentation consistently reaches greater than 95% compliance for all individual vital signs

**Fig. 4:**
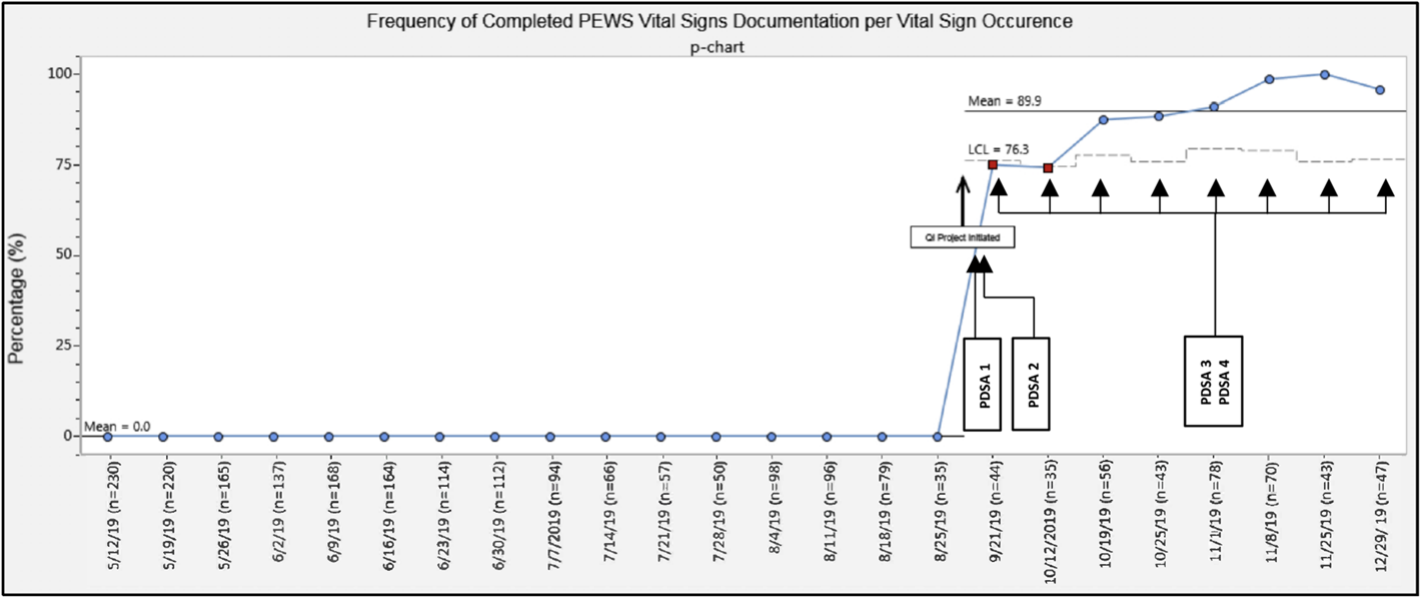
Frequency of completed PEWS vital signs documentation per vital sign occurrence. Figure 4 demonstrates the frequency of complete vital signs documentation (defined as all six vital signs required for PEWS scoring recorded in a vital sign occurrence)

Demographics, admission characteristics, and individual vital sign occurrence were assessed for the 6-month pre-intervention period. A total of 4136 vital sign occurrences were collected in the pre-intervention period. 184 unique patients accounted for 843 total admissions. The majority of encounters resulted in discharge (*n* = 832, 98%), with the remainder transferred for higher level of care (*n* = 8, < 1%) or missing disposition (*n* = 3, < 1%). In the post-intervention period, a total of 8 data audits were completed over the course of 14 weeks. Each data audit reviewed between 35 to 78 vital sign occurrences with the number dependent upon the ward volume on the day of data collection (Table 3) for a total of 438 vital sign occurrences over the course of the post-intervention period (Fig. 2). Fig. 3 demonstrates the frequency of individual vital sign documentation per vital sign occurrence in both the pre-intervention post-intervention data audit periods. During the post-implementation period, there was a substantial improvement in documentation of individual vital signs, with all vital signs being collected with over 95% compliance at the end of the three month post-intervention period. A statistical process control chart (SPC) (Fig. 4) evaluated the frequency of completed PEWS vital signs documentation per vital sign occurrence. In the post-intervention period, frequency of complete PEWS vital sign documentation reached a mean of 89.9% (Fig. 4). The frequency and accuracy of PEWS scores steadily increased during the post-implementation period (Table 3), consistently above 89% in both categories from data audit four onward. Accuracy of PEWS scoring was unable to be assessed during week 1 and 2 of data audits due to challenges with PEWS integration into the existing EMR (PDSA cycle 1) which were resolved by the 3rd week of data auditing (PDSA cycle 2).

## Discussion

Utilizing a partnership approach between physicians and nurses, we collectively developed and implemented a contextually adapted resuscitation program and adapted and operationalized an early warning score system into the ward nursing and physician workflow. Using standard QI methodology including a needs assessment, PDSA cycles for implementation, and SPC charts to plot measurement, we dramatically improved vital sign collection for all vital sign occurences, significantly improved complete vital sign collections (all six vital signs collected during a vital sign occurrence), and sustained frequent and accurate PEWS scoring throughout the post-implementation data auditing period. Previous studies have shown that early warning score tools can accurately identify patients at risk for clinical deterioration in low-resource settings [18], thus providing nurses with objective data to identify patients requiring physician or resident bedside assessments. Our initiative demonstrates a successful approach to implementing a pediatric early warning tool in a low-resource setting, using easily replicatable interventions.

In low- and lower-middle income countries and economies, limitations on resource availability predispose patients to higher morbidity and mortality and contributes to substandard quality of care. In the occupied Palestinian territory, structural determinants of health have led to a fragmented health care system defined by severe resource limitations including health care workforce shortages. Systems-based partnership initiatives, such as our resuscitation and early warning score initiative, support capacity building of the health system to address these limitations. Effective global health partnership interventions should start with effective planning through robust needs assessments. This includes aligning requests and solutions with local health partners, adaptation of external programs to the local context, and assessing for feasibility and sustainability. Incorporating contextual nuances into the education and implementation, such as medication or equipement availability, shift coverage and timing when developing escalation algorithms, PEWS data documentation location, among others, help to ensure uptake and sustainability of programming. From our experience, on-the-ground implementation support and multiple early, rapid PDSA cycles were able to effectively recognize and respond in real time to challenges identified during the period of shoulder-to-shoulder support. Examples of this included ideal location of PEWS documentation within the EMR, which required multiple, rapid iterations as well as a flexible care model. Delays in these cycles could have resulted in delayed or failed ward uptake of the PEWS system.

Our interventions maintained the longitudinal compliance of a PEWS tool by engaging successfully in a partnership consisting of three health-focused organizations. Nursing staff were able to consistently collect all vital signs and score PEWS accurately during the majority of vital sign occurrences. Explanations for the encouraging results are likely due to the tailoring of ward needs to the delivered intervention, the prioritization of physician and nursing education by the ward leadership, buy in from all stakeholders, and supportive ward leadership throughout the implementation process.

The process measures in this study, including the evaluation of the implement of the PEWS *tool* and improving baseline vital signs collection, are a foundational first step to evaluating the effects of PEWS *systems*—the combination of PEWS tool, escalation algorithms, and physician and nursing clinical training—on patient level clinical outcomes. The distinction between PEWS tools and systems is important as PEWS scores are integral in the ability to identify patients at risk for clinical deterioration but cannot function without the ability to alert skilled providers to those patients via escalation algorithms. Previous studies have demonstrated that a modified PEWS tool—a score which retained key elements of traditional PEWS but adjusted for nursing knowledge-base, vital sign limits per hospital standards, and practice variations—decreased clinical deterioration events and PICU utilization in an LMIC oncology inpatient setting when integrated into a PEWS system [19]. Furthermore, given that pediatric cancer patients are at high risk for severe infection during treatments such as chemotherapy, PEWS systems may be of specific importance to identifying, triaging, and responding to this higher risk population. In the pediatric ward setting, an ongoing study to evaluate the PEWS system implementation on clinical outcomes, including time to antibiotics, time to fluid resuscitation, and mortality in patients with elevated PEWS scores, is currently ongoing.

Utilizing PEWS systems for risk stratification of clinically deteriorating patients may also help address patient volume burdens placed on health settings that suffer staff shortages. In the ward, the morning/early afternoon shift relies on two oncologists providing direct patient care to inpatients, outpatients, and the infusion center. During the afternoon/overnight shift, one resident covers all pediatric wards (floor, neonatal intensive care unit, emergency department, and the ward). Given the potential burden of volume of patients a physician or resident may be responsible for, PEWS may provide nurses with an objective triaging tool to efficiently risk stratify and prioritize patients at risk for clinical deterioration, leading to more efficient nursing and physician workforce utilization. Yet, it should be noted that a PEWS system that alerts providers to evaluate patients at the bedside that ultimately do not require interventions could potentially place an unintended strain on ward workforce, which is an important balancing measure in a PEWS system implementation. In our clinical setting, further evaluation of PEWS escalation ‘triggers’ and related clinical interventions should be longitudinally assessed to evaluate for unintended negative consequences of the PEWS intervention.

### Limitations

There are several limitations to our study. Post intervention vital signs and PEWS data relied on random sampling of limited vital signs occurrences, thus potentially overestimating the compliance and accuracy of vital signs and PEWS scores, although the randomized selection of charts to be audited and the variation in days between audits should have minimized the likelihood of this phenomenon. Selection bias in data audits might also have resulted in the assessment of vital sign collection from a limited number of nurses and not reflect departmental practices as a whole. Yet, the random selection of data audit days should limit the potential for this bias. There may also be limitations to generalizability of the findings given the implementation was limited to a pediatric ward.

## Conclusion

Implementation of a PEWS scoring tool into practice in an LMIC pediatric hematology/oncology ward is feasible and can foster effective practice change by improving the frequency of nursing vital sign collection and generate an accurate PEWS scores, which are key process measures for successful implementation of an early warning system. PEWS education, job aids, and frequent QI audits provide robust support and feedback to nurses and physicians to improve and maintain practices in real-time.

## Supplementary Information

**Additional file 1: Supplemental document 1.** Interviews/Focus Groups: MD&RN Demonstrates the questions asked during the needs assessment

**Additional file 2: Supplemental document 2.** Pediatric Early Warning Score-Resource Limited (PEWS-RL) Tool Description of variables used in scoring of PEWS-RL

## Data Availability

The datasets used and/or analyzed during the current study may be available from the corresponding author on reasonable request.

## Abbreviations

BCH GHP: Boston Children’s Hospital Global Health Program
PEWS: Pediatric early warning scores
MOH: Ministry of Health
NGO: Non-governmental organization
oPt: occupied Palestinian territories
LMIC: Low or Lower Middle Income Country
PICU: Pediatric intensive care unit
CRF: Case report form
h/o: Hematology/Oncology
