# Supplementary material for: Implementation of a pediatric early warning score tool in a pediatric oncology Ward in Palestine
Source: BMC Health Serv Res. 2021 Oct 26;21:1159. doi: 10.1186/s12913-021-07157-x (PMC8549265; doi:10.1186/s12913-021-07157-x)
Supplement: Supplementary file 1 — Additional file 1: Supplemental document 1. Interviews/Focus Groups: MD&RN Demonstrates the questions asked during the needs assessment [file 12913_2021_7157_MOESM1_ESM.docx]

*Supplemental Document 1*

**Interviews/Focus Groups: MD & RN**

Interview/Focus Group Questions:

- What is the staffing model?
- What are the most difficult aspects of practicing medicine (ward, hospital, country, etc)?
- What are the biggest concerns around medical practice?
- Changes you’d like to see in the delivery of care/access to care?
- Patient’s perceptions of care?
- What are some barriers to care provision?
- What are some barriers to resuscitation?
- What is the current procedure for recognizing and escalating care?
  - What are the barriers?
- Barriers to communication?
- Barriers to obtaining vital signs?
- Thoughts around feasibility, challenges, benefits of resuscitation training?
- Topics of interest to learn more about in the future?
- Do you feel like an integral member of the care team? Of a resuscitation?
- Any other background topics that are important to discuss?
- What do you feel is a priority to improve patient outcomes/prevent mortality?
- Review of decision to transfer timing and time to transfer to inpatient/perceptions of the transfer process?
- What education have you had so far on assessments? Resuscitation? Emergency medications? Supplies?
- Do staff report understanding of above topics?
