# Supplementary material for: Implementation of a pediatric early warning score tool in a pediatric oncology Ward in Palestine
Source: BMC Health Serv Res. 2021 Oct 26;21:1159. doi: 10.1186/s12913-021-07157-x (PMC8549265; doi:10.1186/s12913-021-07157-x)
Supplement: Supplementary file 2 — Additional file 2: Supplemental document 2. Pediatric Early Warning Score-Resource Limited (PEWS-RL) Tool Description of variables used in scoring of PEWS-RL [file 12913_2021_7157_MOESM2_ESM.docx]

**Pediatric Early Warning Score-Resource Limited (PEWS-RL) Tool**

| **Vital Sign** | **Description** |
| --- | --- |
| Respiratory Rate | - Age-based ranges |
| Respiratory Distress  (Yes or No) | - Age-based ranges |
| Oxygen Requirement  (Yes or No) | - Requirement of oxygen |
| Heart Rate | - Age-based ranges |
| Temperature | - Temperature cutoffs for elevated or low temperatures |
| Mental Status | - AVPU scale - Alert = normal - Responds to pain/voice or unresponsive = abnormal |
| Total | - Each category receives 0 (normal) or 1 (abnormal) point - Total possible score of 6 - An abnormal score is defined as 3 or higher and requires a bedside assessment by a physician |

*For more information please see:
Rosman SL, Karangwa V, Law M, Monuteaux MC, Briscoe CD, McCall N. Provisional Validation of a Pediatric Early Warning Score for Resource-Limited Settings. Pediatrics. 2019;143(5):e20183657. doi:10.1542/peds.2018-3657*
